# Counter-factual mathematics of counterfactual predictive models

**DOI:** 10.3389/fpsyg.2014.00801

**Published:** 2014-08-25

**Authors:** Maria Otworowska, Johan Kwisthout, Iris van Rooij

**Affiliations:** Radboud University Nijmegen, Donders Institute for Brain, Cognition and BehaviourNijmegen, Netherlands

**Keywords:** predictive processing, formal modeling, counterfactual richness, Laplace approximation, sensorimotor contingencies, phenomenology

In order to explain the distinct phenomenology of veridical and non-veridical percepts, Seth (2014) introduces the concept of counterfactual predictions to the Predictive Processing (PP) framework proposed by Clark (2013). The PP framework assumes that the brain generates predictions of its own sensory inputs based on generative models of the world that are learned over time. Seth (2014) proposes to extend this framework by assuming that the brain not only makes predictions of actual sensory inputs, but also of the possible sensory consequences of a variety of possible actions. These so-called counterfactual predictions are presumed to be based on generative models that encode previously learned sensorimotor dependencies. Seth then argues that counterfactually rich generative models can explain why the phenomenology of veridical percepts differs from that of non-veridical percepts, such as arise in synaesthesia.

While Seth deliberately—and understandably, given the aims of his paper—decided to put “the detailed mathematics aside” (Seth 2014, p. 8), we would like to point out that these details become of primary concern when assuming that counterfactual models can encode learned sensorimotor dependencies. The only current candidate formalization of counterfactual predictive processing is given by Friston et al. (2012), work on which Seth says to build. Yet, this particular formalization does not seem to provide the degrees of freedom required to accommodate the counterfactual richness of generative models as envisioned by Seth. The reason is that this formalism is committed to the Laplace assumption: the brain encodes probability distributions as (potentially, multidimensional) Gaussian densities. Friston has consistently defended the Laplace assumption for its neural plausibility and representational efficiency (Friston et al., 2007, 2008; Friston, 2009; Friston et al., 2012). Be that as it may, the Laplace assumption seems to be too restrictive for encoding the distributions corresponding to learned sensorimotor dependencies. We illustrate this point with an example scenario.

Assume one perceives a fruit lying on the table, and it is tilted such that only its bottom is visible. From this perspective it is not possible to tell what type of fruit it is exactly (e.g., it could be an apple or a pear), and hence there is ambiguity about the counterfactual predictions that apply about the sensory consequences of possible actions that can be performed on the fruit. For instance, it could be that if one were to grasp the bottom of the fruit and turn it, one would see that the other side of the fruit is round (e.g., if it were an apple), or alternatively, one may see that the fruit is cone shaped (e.g., if it were a pear). Similarly, it could be that if one were to grasp the non-visible top of the fruit that the aperture of the fingers will be relatively large when the fingers touch the surface (e.g., if it were an apple), or alternatively, relative small (e.g., if it were a pear). In our world, fruits are often round (e.g., when they are apples), sometimes cone shaped (e.g., when they are pears), but rarely do fruits have shapes in-between round and cone. Given these relative frequencies of fruit shapes, learned sensorimotor contingencies will lead to probability densities for counterfactual predictions that are multimodal; e.g., have a peak around “round” and a peak around “cone,” but lower probabilities for shapes in between (see Figure 1 for an illustration).^1^

**Figure 1:**
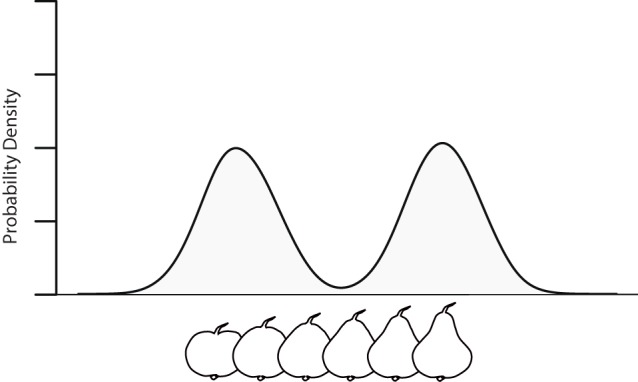
**Probability density for a dimension for which we can distinguish the round shapes characteristic of an apple from the cone shape characteristic of a pear, conditioned on (i) the sensory input generated by the bottom view of the fruit, (ii) the prior density describing the frequency of different shaped fruits in the world, and (iii) the hypothetically performed action of, say, grasping the fruit from the bottom and turning it so as to view it from the side**.

Note that in our example scenario the Laplace assumption made by Friston et al. (2012) is violated. Given that we can distinguish between the sensory consequences of acting upon round shapes (such as are characteristic of apples) and cone shapes (such as are characteristic of pears) there must exist at least one dimension—and possibly multiple dimensions—in the multidimensional density that constitutes the counterfactual generative model with the property that there is a range of values representing shapes in-between the value on that dimension for “round” and the value for “cone” (otherwise the value of “round” and “cone” would be equal for all dimensions, making it impossible for us to tell them apart). The Laplace assumption would imply that the probability of each of these intermediate values would need to be at least as high as the probability of the values corresponding to “round” or “cone” shape (otherwise the density would be multimodal, and hence not Gaussian). Yet, as illustrated in our scenario, this is arguably not true for fruits in our world.

Given the above considerations, the existing formalization of counterfactual PP seems to lack the degrees of freedom required for counterfactual PP explanations of phenomenological experience as envisioned by Seth.^2^ This does not mean that such a formalization is unattainable, but it may look substantially different from the one presumed by Seth. For instance, there exist mixture models that can perform inferences on the types of mixtures of Gaussians illustrated in our Figure 1, and contrary to Friston (2009), it has been argued that these mixture models have neural (Pecevski et al., 2011) and representational (Gershman et al., 2009) plausibility. Yet, the integration of these models in the PP framework is highly non-trivial, because simple formalizations of central concepts in PP that hold under the Laplace assumption (such as “precision” defined as $\frac{1}{\sigma^{2}}$) do not straightforwardly translate to multimodal distributions. Hence, Seth's proposal looks promising, but to reach its full explanatory potential, work urgently needs to be done on the mathematical formalization of his theory.

## Conflict of interest statement

The authors declare that the research was conducted in the absence of any commercial or financial relationships that could be construed as a potential conflict of interest.

## Footnotes

^1^To be clear, we do not mean to suggest that in ambiguous cases such as these, that humans experience the ambiguity between “seeing an apple” vs. “seeing a pear” when presented with an ambiguous view from the bottom. For all we know, no such ambiguity is ever experienced. Our point is merely that if counterfactual predictions are based on learned veridical sensorimotor dependencies, then the densities corresponding to those predictions need to capture the actual frequencies of those dependencies in the world, which can be multimodal distributions.

^2^We note that this concern is not specific to Seth's theory, and may in fact apply more broadly to other PP explanations in the current literature. For instance, the prominent account of binocular rivalry as put forth by (Hohwy et al., 2008) seems to also appeal to multimodal distributions within a PP framework (see their Figure 5, p. 693).
